# Positioning Performance Limits of GNSS Meta-Signals and HO-BOC Signals

**DOI:** 10.3390/s20123586

**Published:** 2020-06-25

**Authors:** Lorenzo Ortega, Daniel Medina, Jordi Vilà-Valls, François Vincent, Eric Chaumette

**Affiliations:** 1 Telecommunications for Space and Aeronautics Lab (TéSA), 31500 Toulouse, France; 2 Institute of Communications and Navigation, German Aerospace Center (DLR), 17235 Neustrelitz, Germany; Daniel.AriasMedina@dlr.de; 3 Institut Supérieur de l’Aéronautique et de l’Espace, University of Toulouse, 31055 Toulouse, France; Francois.Vincent@isae-supaero.fr (F.V.); Eric.CHAUMETTE@isae-supaero.fr (E.C.)

**Keywords:** GNSS, Cramér–Rao bound, time-delay and phase ML estimation, SPP and RTK positioning, precise positioning, GNSS meta-signals, high-order BOC signals

## Abstract

Global Navigation Satellite Systems (GNSS) are the main source of position, navigation, and timing (PNT) information and will be a key player in the next-generation intelligent transportation systems and safety-critical applications, but several limitations need to be overcome to meet the stringent performance requirements. One of the open issues is how to provide precise PNT solutions in harsh propagation environments. Under nominal conditions, the former is typically achieved by exploiting carrier phase information through precise positioning techniques, but these methods are very sensitive to the quality of phase observables. Another option that is gaining interest in the scientific community is the use of large bandwidth signals, which allow obtaining a better baseband resolution, and therefore more precise code-based observables. Two options may be considered: (i) high-order binary offset carrier (HO-BOC) modulations or (ii) the concept of GNSS meta-signals. In this contribution, we assess the time-delay and phase maximum likelihood (ML) estimation performance limits of such signals, together with the performance translation into the position domain, considering single point positioning (SPP) and RTK solutions, being an important missing point in the literature. A comprehensive discussion is provided on the estimators’ behavior, the corresponding ML threshold regions, the impact of good and bad satellite constellation geometries, and final conclusions on the best candidates, which may lead to precise solutions under harsh conditions. It is found that if the receiver is constrained by the receiver bandwidth, the best choices are the L1-M or E6-Public Regulated Service (PRS) signals. If the receiver is able to operate at 60 MHz, it is recommended to exploit the full-bandwidth Galileo E5 signal. In terms of robustness and performance, if the receiver can operate at 135 MHz, the best choice is to use the GNSS meta-signals E5 + E6 or B2 + B3, which provide the best overall performances regardless of the positioning method used, the satellite constellation geometry, or the propagation conditions.

## 1. Introduction

Global Navigation Satellite Systems (GNSS) are the main source of position, navigation, and timing (PNT) information in several engineering fields. The main advantages are their global coverage and that the satellite-based infrastructure is already deployed and maintained by governmental institutions. The latter allows the user to exploit GNSS freely and the ability to design both mass-market and dedicated receivers. These remarkable advantages will make GNSS a key player in the next-generation intelligent transportation systems and several safety-critical applications. However, even if the navigation research community has been developing positioning methodologies for decades, there are still several limitations that may limit the use of GNSS in the most stringent applications, i.e., lane-level precision for autonomous driving in highly populated cities with harsh propagation conditions. One of the key open problems is how to achieve precise PNT solutions under harsh environments, i.e., affected by multipath, deep fading, signal blockage, or non-line-of-sight (NLOS) conditions. Using standard GNSS signals, it is known that code-based techniques (i.e., only relying on the time-delay estimation between the receiver and a set of visible satellites) do not provide precise PNT information. The standard way to provide such precise navigation capabilities is by exploiting carrier phase information. Indeed, this measurement is linked to the wavelength, which is much smaller than the baseband signal resolution (i.e., for a legacy Global Positioning System (GPS) L1-C/A signal, the wavelength is 19.4 cm, while the baseband signal resolution is around 300 m). The two main solutions are precise point positioning (PPP) [1] and real-time kinematic (RTK) positioning [2]. However, the main problem of these techniques is that they are very sensitive to the quality of phase observables, i.e., it is unlikely that they provide a robust solution under harsh propagation conditions, at least exploiting standard GNSS signals. Therefore, in order to provide robust and precise solutions, new alternatives must be accounted for. A possible alternative is to robustify the signal processing, for instance resorting to outlier mitigation techniques [3,4]. Another option is to increase the receiver complexity and exploit large bandwidth signals, which allow obtaining a better (i.e., with respect to standard signals) baseband resolution, and therefore more precise code-based observables. The latter can be achieved by using (i) high-order binary offset carrier (HO-BOC) modulations or (ii) GNSS meta-signals, which is the combination of two GNSS signals at different frequency bands as a single signal.

It is a fact that there is an increasing interest in the topic of GNSS meta-signals. The authors are not aware if this is the case on the industry side, but on the academic side, the European Space Agency (ESA) is funding (or has recently funded) projects related to the study of HO-BOC signals and GNSS meta-signals, in order to obtain more precise code-based pseudoranges [5,6,7]. For instance, one of the main goals of the latest one [7] is to assess if such GNSS meta-signals are an option to achieve sub-meter- or decimeter-level positioning in harsh propagation conditions (i.e., multipath and urban canyons), mainly because carrier phase-based positioning techniques are known to fail in such challenging scenarios as the phase ambiguity resolution needs precise phase observables. However, there are very few contributions discussing the concept of meta-signals [8,9]; therefore, before considering realistic harsh propagation conditions, an important missing point in the literature is the characterization of the ultimate positioning performance limits of such signals (i.e., resorting to fundamental estimation performance bounds), which is the goal of this article. In that perspective, in a recent contribution, we provided the derivation of a compact joint time-delay and phase estimation Cramér–Rao bound (CRB) and the time-delay ML estimation (MLE) performance limits of different GPS and Galileo signals, including two Galileo-based meta-signals [10]. However, no further discussion was provided on the impact on the position estimation, considering both code and phase observables.

With respect to [10], the main contributions of this article are:

- An overview of the GNSS meta-signals, including combinations of GPS, BeiDou, and Galileo signals.
- A comparison of GNSS meta-signals with a set of representative HO-BOC signals.
- A short discussion of the use of very large bandwidth meta-signals.
- The impact of the time-delay MLE behavior (related to the secondary peaks of the autocorrelation function (ACF)) on the phase MLE and the convergence to the phase CRB.
- The performance limits of code-based SPP considering the use of GNSS meta-signals, for both good and bad satellite geometries; results for HO-BOC-based SPP are also provided.
- The application of GNSS meta-signals in the context of precise carrier phase-based RTK positioning, to assess whether exploiting the signal phase information is worthwhile for large bandwidth signals; again, both good and bad satellite geometries are considered; for completeness, the results for HO-BOC-based RTK are also provided.
- Recommendations on the best candidates that may lead to precise PNT under harsh conditions.

Notice that the goal of this article is to obtain the asymptotic positioning performance limits, that is the estimation errors only related to the GNSS signal and not to external errors. Therefore, we do not consider ionospheric/tropospheric delays, orbital or satellite clock errors, or environment-specific effects such as multipath [11]. Moreover, these external errors are the same whatever the processing. Then, we are interested in the asymptotic region of the MLE, which is characterized with respect to (w.r.t.) the signal-to-noise (SNR) ratio and considering only the thermal noise. Refer for instance to [12] for the analysis of the specific impact of external errors and different types of corrections.

## 2. GNSS Signal Model, Meta-Signals, and HO-BOC Signals

### 2.1. Signal Model

The GNSS signals broadcast by the different satellite constellations are typically built as a multilayer structure: (i) a low rate navigation message, encoded as binary phase-shift keying (BPSK) bits; (ii) a fast rate ranging code, so-called pseudo-random noise (PRN) code, with good autocorrelation and cross-correlation properties in order to allow individual satellite signals’ processing (i.e., quasi orthogonality); (iii) a subcarrier that modulates the PRN code and shapes the autocorrelation function (ACF), i.e., no subcarrier is employed for the legacy GPS L1-C/A signal or binary offset carrier (BOC)-type subcarriers in modernized GPS and some Galileo signals; (iv) a carrier that is used to allocate the complete signal into the corresponding frequency. Notice that the signal may have data bits or not, depending on whether it belongs to a data component or a pilot component. In the sequel, and without loss of generality, we do not account for navigation data bits within the observation time.

In general, the signal at the receiver antenna is the superposition of a set of signals at different frequency bands, plus environmental effects such as multipath and/or interferences. The latter two effects are out of the scope of this contribution, and because of the quasi orthogonal PRN code design, we can focus on one of these signals to define the model to be exploited. Therefore, we consider the transmission of a band-limited GNSS signal c(t) (bandwidth *B*), which contains both the PRN and subcarrier, over a carrier frequency $f_{c}$ ($\lambda_{c}=c/f_{c}$), from a transmitter (satellite) *T* to a receiver *R*. If *T* and *R* follow a relative uniform radial movement, then the *T*-to-*R* distance, $p_{TR}\left(t\right)$, can be approximated by a first-order distance-velocity model [13,14,15,16],

(1) $$\begin{array}{cc} & ∥p_{TR}\left(t\right)∥≜∥p_{R}\left(t\right)-p_{T}\left(t-\tau \left(t\right)\right)∥=c\tau \left(t\right)≃d\;+\;vt,\\ \; & \tau \left(t\right)≃\tau \;+\;bt,\;\tau =\frac{d}{c},\;b=\frac{v}{c},\;c=299,792,458\;m/s, \end{array}$$

where *d* is the *T*-to-*R* relative radial distance and *v* is the *T*-to-*R* relative radial velocity. Note that the previous formula is characterized by a time-delay $\left(\tau \right)$ due to the propagation path and a dilation (1−b) induced by the Doppler effect. The complex analytic signal at the output of the receiver’s antenna can be written as:

(2) $$x_{A}\left(t\right)=\alpha_{R}c\left(\left(1-b\right)\left(t-\tau \right)\right)e^{j2\pi f_{c}\left(1-b\right)t}e^{-j2\pi f_{c}\tau }\;+\;n_{A}\left(t\right),$$

with *e* the Euler number (exponential function), $n_{A}\left(t\right)$ a zero-mean white complex Gaussian noise, and where the gain $\alpha_{R}$ depends on the transmitted signal power, the transmitter/receiver antenna gains, polarization vectors, and the radial distance between T and R [17,18]. Notice that the following the standard narrowband assumption (2) can be approximated as [19,20]:

(3) $$x_{A}\left(t\right)\approx \alpha_{R}c\left(t-\tau \right)e^{j2\pi f_{c}\left(1-b\right)t}e^{-j2\pi f_{c}\tau }\;+\;n_{A}\left(t\right),$$

and because we are only interested in the processing of time-delay and phase information, (3) can be further simplified considering that during the observation time (i.e., 1 ms), both *T* and *R* are static, that is their respective positions are constant $p_{T}\left(t\right)=p_{T}$ and $p_{R}\left(t\right)=p_{R}$. In that perspective, the propagation delay $\tau \left(t\right)$ is constant $\tau \left(t\right)=\tau =\frac{∥p_{TR}∥}{c}$, and the baseband output of the receiver’s Hilbert filter is given by:

(4) $$x\left(t\right)\approx \alpha c\left(t-\tau \right)\;+\;n\left(t\right),$$

with n(t) a complex white Gaussian noise within the filter bandwidth with unknown variance $\sigma_{n}^{2}$ and $\alpha =\alpha_{R}e^{-j2\pi f_{c}\tau }$. The discrete vector signal model is built from $N=N_{2}-N_{1}\;+\;1$ samples at $T_{s}=\frac{1}{F_{s}}$, where $F_{s}$ is the sampling frequency:

(5) $$\begin{array}{cc} x & =\alpha c\left(\tau \right)\;+\;n,\\ x & ={\left(x\left(N_{1}T_{s}\right),\ldots ,x\left(N_{2}T_{s}\right)\right)}^{⊤},\\ n & ={\left(n\left(N_{1}T_{s}\right),\ldots ,n\left(N_{2}T_{s}\right)\right)}^{⊤},\\ c\left(\tau \right) & ={\left(c\left(N_{1}T_{s}-\tau \right),\ldots ,c\left(N_{2}T_{s}-\tau \right)\right)}^{⊤}, \end{array}$$

where $n∼\mathrm{CN}\left(0,\sigma_{n}^{2}I_{N}\right)$. Since the transmitter/receiver antenna gains and polarization vectors are in general unknown, α is assumed to be an unknown complex parameter as well [18,21,22,23,24]. Thus, the unknown deterministic parameters [25] can be gathered in a vector $\underline{\epsilon }={\left(\sigma_{n}^{2},\tau ,\alpha ,\alpha^{*}\right)}^{⊤}$, where $\alpha^{*}$ is the complex conjugate of α. The model in (5) can be reparametrized to make explicit the phase parameter,

(6) $$x=\rho c^{\prime }\left(\theta \right)\;+\;n,\;c^{\prime }\left(\theta \right)=c\left(\tau \right)e^{j\varphi },\;\rho \in R^{\;+\;},\theta^{⊤}=\left(\varphi ,\tau \right),$$

and then, the unknown deterministic parameters are $\underline{\epsilon }={\left(\sigma_{n}^{2},\rho ,\theta^{⊤}\right)}^{⊤}$; ρ is the amplitude of the signal; and θ gathers the unknown phase φ and time-delay τ.

### 2.2. Generalized AltBOC

The GNSS meta-signal is a basic concept that consists of processing two GNSS signals transmitted at two different carrier frequencies as a single GNSS signal [8,9]. In order to process both signals jointly, the so-called alternate binary offset carrier (AltBOC) modulation or the alternate linear offset carrier (AltLOC) modulation [26] can be used, the goal being to express the two signals as a single one. The reader can refer to [27] and the previous references for a more exhaustive definition of the generalized alternate binary offset carrier (AltBOC) modulation and the corresponding spectral and ACF properties. The idea to process two GNSS signals located at two different bands jointly comes from the proposal made by the the Galileo Signal Task Force group in the year 2000. In the original proposal, an AltBOC signal was considered to transmit two independent signals in two separate bands using a unique high power amplifier (HPA). An AltLOC signal was also studied at the time, but it was quickly dropped because its envelope was not constant. Notice that a constant envelope signal at the transmitter is interesting in order to enhance the HPA efficiency. However, as was shown in [26], once the transmitter filters the intermodulation products and the harmonics of the AltBOC signal, the transmitted signal yields to an AltLOC modulation. Therefore, two different GNSS signals can be jointly processed at the receiver as a single AltLOC-modulated signal.

The easiest form of AltLOC-modulated signal is the one where two independent PRN codes are multiplexed. Let us define the subcarrier with cosine and sine phasing as $SC_{cos}\left(t\right)=\cos\left(2\pi F_{sub}t\right)$ and $SC_{sin}\left(t\right)=\sin\left(2\pi F_{sub}t\right)$, respectively, where $F_{sub}$ represents the subcarrier frequency. Then, we can build the single side band (SSB) subcarrier $SC_{SSB}$ and its conjugate $SC_{SSB}^{*}$ as,

(7) $$\begin{array}{c} SC_{SSB}\left(t\right)=\frac{1}{\sqrt{2}}\left(SC_{cos}\left(t\right)\;+\;j\cdot SC_{sin}\left(t\right)\right)\;;\;SC_{SSB}^{*}\left(t\right)=\frac{1}{\sqrt{2}}\left(SC_{cos}\left(t\right)-j\cdot SC_{sin}\left(t\right)\right) \end{array}$$

where *j* represents the imaginary part. The two-code AltLOC can be defined as,

(8) $$\begin{array}{cc} c(t) & =c_{A}\left(t\right)SC_{SSB}^{*}\left(t\right)\;+\;c_{B}\left(t\right)SC_{SSB}\left(t\right)=\left[c_{A}\left(t\right)\;+\;c_{B}\left(t\right)\right]SC_{cos}\left(t\right)\;+\;j\cdot \left[c_{B}\left(t\right)-c_{A}\left(t\right)\right]SC_{sin}\left(t\right) \end{array}$$

where $c_{A}\left(t\right)$ and $c_{B}\left(t\right)$ represent the GNSS signals at the low (*A*) and high (*B*) frequency bands, respectively. Note from Equation (8) that the codes $c_{A}\left(t\right)$ and $c_{B}\left(t\right)$ are not required to have the same chip rate. We use the notation AltLOC(p,q,w,b) to represent the AltLOC receiver signal. Thus, given the fundamental frequency $f_{0}=1.023$ MHz, *p* is the real number used to represent the subcarrier frequency $F_{sub}$ through the equality $F_{sub}=pf_{0}$; *q* is the real number used to represent the lower code chip rate $f_{c,A}$ through the equality $f_{c,A}=qf_{0}$; *w* is the real number used to represent the upper code chip rate $f_{c,B}$ through the equality $f_{c,B}=wf_{0}$; and *b* is the real number used to represent the receiver bandwidth BW through the equality $BW=bf_{0}$.

### 2.3. GNSS Meta-Signals

In the previous contribution [27], we defined the original Galileo E5 signal, which uses an AltBOC(15,10), and the corresponding combinations with the Galileo E6 band signal, E5B + E6 and E5A + E6 meta-signals, being modulated as a single AltBOC(35,10,5) and AltBOC(50,10,5). These signals can be alternatively defined using the previous AltLOC modulation. In addition, several other signal combinations using individual GPS, Galileo, and BeiDou signals can be considered. The complete set of GNSS meta-signals considered in this article includes the following combinations:

- Galileo E5 = E5A + E5B, generated as an AltBOC(15,10) [28] and taken as a reference.
- GPS L5 + L2C, generated through an AltLOC(25,10,1,75). The central frequency of this meta-signal is located at $f_{c}=1175f_{0}$.
- Galileo E5B + E6, generated through an AltLOC(35,10,5,112). The central frequency of this meta-signal is located at $f_{c}=1215f_{0}$.
- Galileo E5A + E6, generated through an AltLOC(50,10,5,132). The central frequency of this meta-signal is located at $f_{c}=1200f_{0}$.
- Galileo E5 + E6, generated through an AltLOC(42.5,$C_{E5}\left(t\right)$,5,132). $C_{E5}\left(t\right)$ represents the full-bandwidth Galileo E5 signal. The central frequency of this meta-signal is located at $f_{c}=1207.5f_{0}$.
- BeiDou B2A + B3, generated through an AltLOC(45,10,10,120). The central frequency of this meta-signal is located at $f_{c}=1195f_{0}$.
- BeiDou B2 + B3, generated through an AltLOC(37.5,$C_{B2}\left(t\right)$,10,125). $C_{B2}\left(t\right)$ represents the full-bandwidth BeiDou B2 signal, characterized by an AltBOC(15,10) modulation [29,30]. The central frequency of this meta-signal is located at $f_{c}=1202.5f_{0}$.

**Figure 1:**
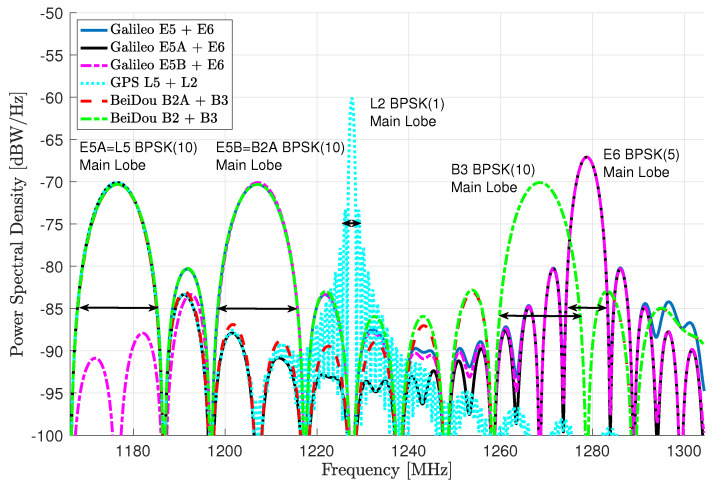
PSD for the different GNSS meta-signals.

Notice that for all the signals, we only consider the corresponding pilot components, i.e., E5 is generated as E5AQ + E5BQ or the combination of E5B and E6 is generated as E5BQ + E6B. For completeness, we provide the different meta-signals power spectral density (PSD) in Figure 1, where we make explicit the individual signals main lobes: E5A, L5, E5B, B2A, and B3 signals using a BPSK(10) (i.e., a BPSK with a chip rate 10 times faster than the one of the GPS L1-C/A), E6 BPSK(5), and L2 BPSK(1) signal.

**Figure 2:**
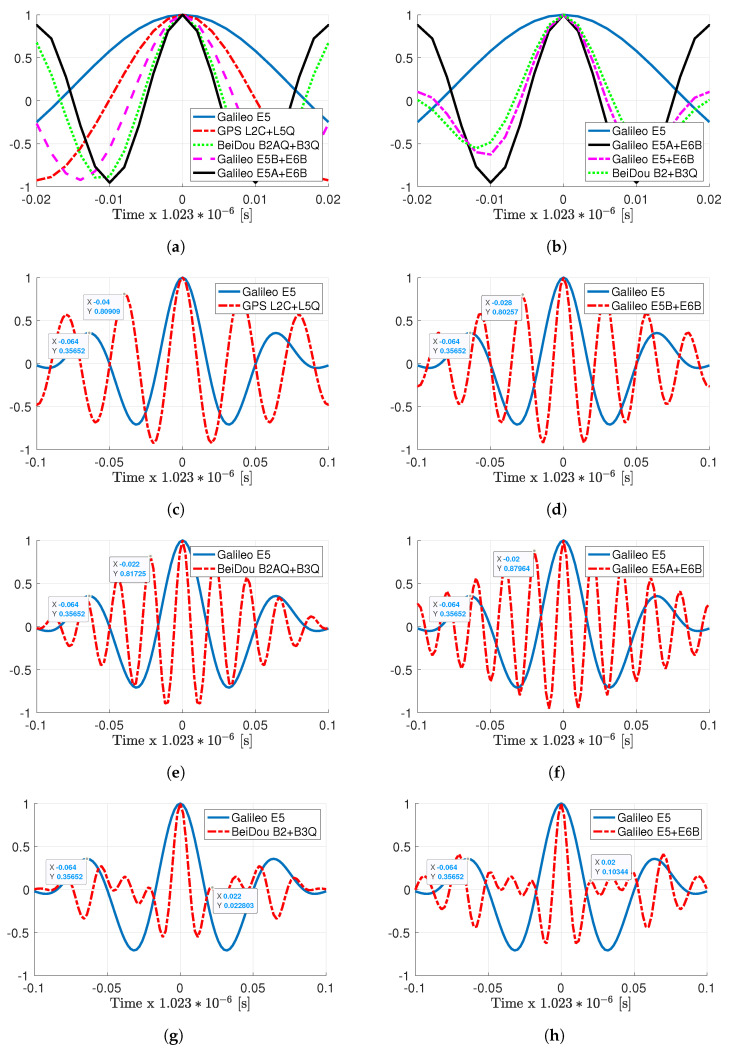
(**a**) Main ACF peak comparison for different meta-signals w.r.t. E5: L2C + L5, B2A + B3, E5B + E6 and E5A + E6. (**b**) Main ACF peak comparison for different meta-signals w.r.t. E5 and E5A + E6: Beidou B2 + B3 and Galileo E5 + E6. The comparison of each individual meta-signal ACF with the Galileo E5 is given in the remaining subplots. (**c**) GPS L2C + L5Q, (**d**) Galileo E5B + E6B, (**e**) BeiDou B2AQ + B3Q, (**f**) Galileo E5A + E6B, (**g**) BeiDou B2 + B3Q and (**h**) Galileo E5 + E6B. (i.e., in all plots, the vertical axis represents the normalized autocorrelation). The magnitude and position of the first secondary peak are given for completeness.

In addition and because it is difficult to extrapolate the behavior of the different signals from their PSD in Figure 1, we show the corresponding ACF in Figure 2. In the two top subplots (a) and (b), we show the comparison of the main ACF peak, where as expected, a wider PSD implies a narrower ACF. What is not evident to guess from the PSD is the shape of the different ACFs in Subplots (c)–(h). In the “standard” signal combinations, the L2 + L5, B2A + B3, E5B + E6, and E5A + E6, narrower main ACF peak implies higher secondary peaks and closer to the main one. The values and position of these secondary peaks are also given for completeness. As will be discussed in the results, these secondary peaks are one of the main drivers of the estimators’ performance. Remarkably, the combinations with the full-bandwidth B2 and E5 signals, that is the combination of AltBOC(15,10) with the signals in the B3 and E6 bands, respectively, exhibit much lower secondary peaks. Despite their nice ACF shape, as far as the authors’ knowledge, these combinations have never been discussed in the literature.

### 2.4. Other Large Bandwidth GPS, Galileo, and BeiDou Signal Combinations

**Figure 3:**
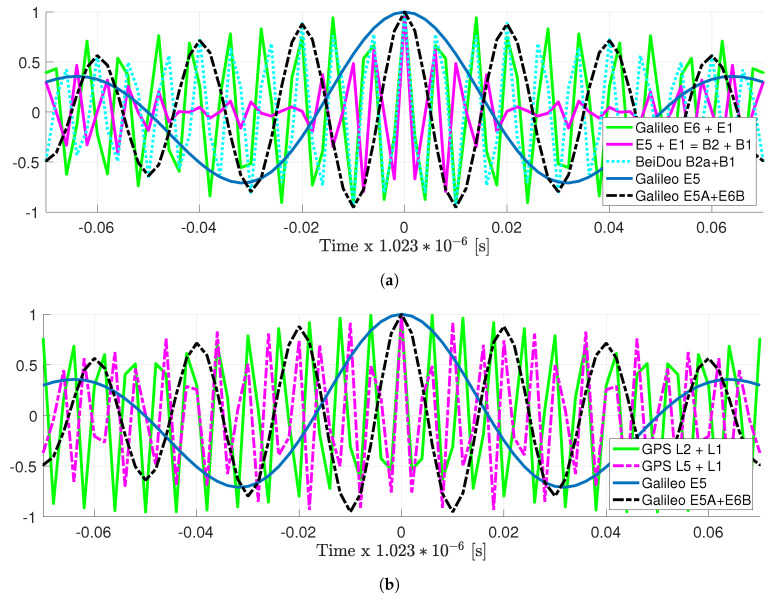
ACF for other very large bandwidth Galileo, BeiDou (**a**) and GPS (**b**) signal combinations, (i.e., in both plots, the vertical axis represents the normalized autocorrelation). For comparison the Galileo E5 ACF and the Galileo E5A + E6 meta-signal ACF are also shown.

Notice that the previous signal combinations considered signals in the L5/E5, E6, and L2 bands. The main goal of such combinations is to have a larger bandwidth and narrower ACF main peak, which in turn may lead to better time-delay estimation performance, and therefore a better position estimation. Since the goal is to have a narrower ACF, one may seek to exploit other signal combinations with the GPS, BeiDou, and Galileo signals in the L1/E1/B1 band. The ACFs for these large bandwidth meta-signals are summarized in Figure 3: GPS L2 + L1, GPS L5 + L1, Galileo E6 + E1, Galileo E5 + E1, BeiDou B3 + B1, and BeiDou B2 + B1.

First, notice that as expected, the main ACF peak is narrower than the narrower Galileo E5A + E6B combination previously considered. However, the price to be paid is larger secondary peaks, which are also closer to the main one. This is an effect that can also be seen in Figure 2c–f, and as already stated, it is a critical point to take carefully into account. Indeed, large secondary peaks must be avoided to minimize the effect of possible false locks. As will be discussed in the rest of the article and being especially clear for the E5A + E6 signal, these possible false locks have a strong impact on the ML behavior (i.e., on the ML threshold, and therefore on the optimal receiver operation point). This is the main reason why these large bandwidth meta-signals are not useful in practice because even if in the asymptotic regime, the time-delay estimation performance may be better, the SNR needed to achieve such a regime is not worth exploiting these signals. In addition, in terms of computational complexity, notice that such combinations need a bandwidth in the order of 400 MHz, exceeding the real-time processing capabilities. Therefore, these combinations were briefly introduced for completeness, but are not taken into account in this contribution.

### 2.5. On the ACF of High-Order BOC Signals

As has been previously stated, an option to increase the code-based observables’ precision is the use of single-band HO-BOC signals. Note that the BOC modulation is generally denoted BOC(p,q), where *p* refers to the sub-carrier frequency $f_{sc}=p\cdot 1.023$ MHz (i.e., $sc\left(t\right)=sign\left[\sin\left(2\pi f_{sc}t\right)\right]$ with sign the sign function) and *q* to the ranging code frequency $f_{c}=q\cdot 1.023$ MHz [31]. In GPS, an HO-BOC modulation is used in the modern military M signal, i.e., L1-M. In Galileo, the Public Regulated Service (PRS) signal in the E1 band uses also an HO-BOC subcarrier, as well as the corresponding PRS in the E6 band. In the case of BeiDou, the B1A HO-BOC-modulated signal is defined as an authorized service. It is important to notice that even if we provide the discussion and results considering these types of modulation, which are useful to benchmark the interest of GNSS meta-signals, the HO-BOC-modulated signals-in-space available are all regulated/military signals. This implies that these signals are not and will not be available in mass-market receivers.

In this article, we consider the following representative set of HO-BOC signals:

- GPS L1-M, BOCsin(10,5)
- Galileo E1 PRS, BOCcos(15,2.5)
- Galileo E6 PRS, BOCcos(10,5)
- BeiDou B1A, BOCcos(14,2)

**Figure 4:**
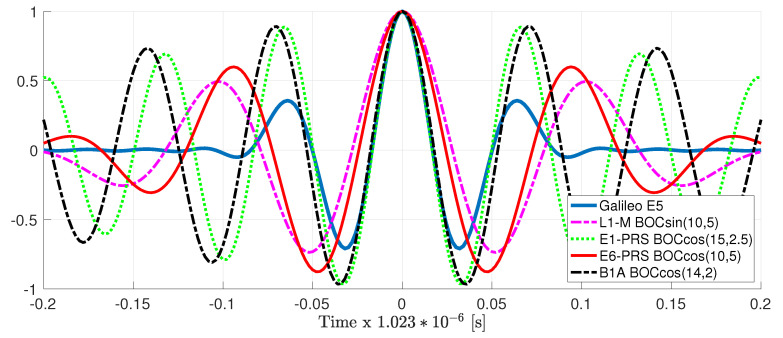
ACF for different high-order (HO)-BOC signals (i.e., the vertical axis represents the normalized autocorrelation). For comparison the Galileo E5 ACF is also shown.

The corresponding ACF for these four signals is depicted in Figure 4 and compared to the Galileo E5 ACF. Notice that the ACF shape of the E1-PRS BOCcos(15,2.5) and B1A BOCcos(14,2) is very similar to the one for the Galileo E5; therefore, we can expect a similar achievable performance (i.e., CRB and asymptotic MLE performance). In terms of the MLE behavior, these two signals have much larger ACF secondary peaks, which will certainly have an impact on the MLE convergence, as will be discussed in the results. Regarding the L1-M and E6-PRS, the ACF shape is also similar, with a slightly wider main peak w.r.t. Galileo E5 and secondary peaks that are larger, but further apart. Therefore, one can expect the convergence not to be degraded and a slightly worse achievable performance compared to the Galileo E5.

Time-delay and phase MLE performance results for the four HO-BOC signals are provided in Section 4. The corresponding SPP and RTK positioning results, considering a good satellite geometry, are discussed in Section 7. It is important to notice that, due to the construction of the signal, the bandwidth needed to process these HO-BOC signals will always be lower than the one needed for the Galileo E5 or the different meta-signals.

## 3. GNSS Receiver Signal Processing

### 3.1. GNSS ML Baseband Signal Processing and Delay/Phase CRB

The final goal of a GNSS receiver is to obtain position, velocity, and time (PVT) estimates. Therefore, from am ML perspective and taking into account that the received signal is the superposition of a set of signals related to the same receiver position, the optimal solution is given by the so-called direct position estimation (DPE) [32,33]. The main disadvantage is that DPE implies solving a high-dimensional minimization problem, which turns into a high computational complexity. Even if DPE approaches are known to provide better position estimates under certain conditions, it has been recently shown that the traditional two-step approach (synchronization + multilateration) is asymptotically optimal [34]. Therefore, in this contribution, we are only interested in the latter, which implies the individual processing of satellite signals thanks to the quasi orthogonality of PRN codes. The goal of the first stage of the receiver is to construct a set of observables for each satellite in view. In our case, this set includes code and phase observables, which are directly linked to the time-delay and phase MLE. Considering the signal model (6), the time-delay and phase MLE are defined as [24]:

(9) $$\begin{array}{cc} \; & \hat{\tau }=\arg\max_{\tau }\left\{{\left|{\left(c{\left(\tau \right)}^{H}c\left(\tau \right)\right)}^{-1}c{\left(\tau \right)}^{H}x\right|}^{2}\right\}, \end{array}$$

(10) $$\begin{array}{cc} \; & \hat{\varphi }\left(\hat{\tau }\right)=\arg\left\{{\left(c{\left(\hat{\tau }\right)}^{H}c\left(\hat{\tau }\right)\right)}^{-1}c{\left(\hat{\tau }\right)}^{H}x\right\}, \end{array}$$

where arg is the argument operator, max is the maximum operator, ${\left(\cdot \right)}^{H}$ is the Hermitian operator, and $\left|\cdot \right|$ is the Euclidean norm. Then, the phase is estimated as the argument of the cross-ambiguity function evaluated at the time-delay MLE. The study of these MLEs is useful to determine the value of the SNR at the output of the matched filter, which allows reaching the CRB, because such estimators are known to be asymptotically efficient (e.g., in the high SNR regime) for the conditional signal model of interest [35,36].

It is also fundamental to have the corresponding CRB, which for the time-delay and phase estimation problem of interest was recently derived in a compact closed-form for a generic band-limited signal in [10]. Such a CRB is very convenient because it only depends on the signal sample, and it is summarized in the sequel for completeness. Considering the joint time-delay and phase $\theta^{⊤}=\left(\varphi ,\tau \right)$ estimation resorting to Model (6), the CRB is given by [37]:

(11) CRBτ∣ϵ_=12SNRout1Fs2cHVccHc−ImcHΛccHc2=realsignal12SNRout1Fs2cHVccHc,

(12) CRBφ∣ϵ_=12SNRout1+ImcHΛccHc2cHVccHc−ImcHΛccHc2=realsignal12SNRout,

where Im represents the imaginary operator, ${\mathrm{SNR}}_{\mathrm{out}}=\frac{{\left|\alpha \right|}^{2}E}{\left(\sigma_{n}^{2}/F_{s}\right)}=\frac{{\left|\alpha \right|}^{2}}{\sigma_{n}^{2}}c^{H}c$, and E the energy of the signal. Λ and V are defined as (for $N_{1}\leq n,n^{\prime }\leq N_{2}$):

(13) $$\begin{array}{cc} \; & {\left(V\right)}_{n,n^{\prime }}=\left|\begin{array}{c} n^{\prime }\neq n:{\left(-1\right)}^{\left|n-n^{\prime }\right|}\frac{2}{{\left(n-n^{\prime }\right)}^{2}}\\ n^{\prime }=n:\frac{\pi^{2}}{3} \end{array}\right.\;;\;{\left(\Lambda \right)}_{n,n^{\prime }}=\left|\begin{array}{c} n^{\prime }\neq n:\frac{{\left(-1\right)}^{\left|n-n^{\prime }\right|}}{\left(n-n^{\prime }\right)}\\ n^{\prime }=n:0 \end{array}\right.. \end{array}$$

### 3.2. GNSS Code and Phase Observables

From the individual satellite delay and phase MLEs and after demodulating the navigation data message, the receiver obtains a set of code and phase observables for each satellite in view. Disregarding ionospheric and tropospheric delays, as well as other sources of error, for the ith satellite, we write,

(14) $$\begin{array}{cc} {\hat{\varrho }}_{i}\; & =\left|\right|p_{T_{i}}-p_{R}\left|\right|\;+\;c\left(\delta t_{r}-\delta t_{i}\right)\;+\;\epsilon_{\varrho ,i}, \end{array}$$

(15) $$\begin{array}{cc} {\hat{\Phi }}_{i}\; & =\left|\right|p_{T_{i}}-p_{R}\left|\right|\;+\;c\left(\delta t_{r}-\delta t_{i}\right)\;+\;\lambda_{c}N_{i}\;+\;\epsilon_{\Phi ,i}, \end{array}$$

where $\left|\right|p_{T_{i}}-p_{R}\left|\right|=\sqrt{{\left(x_{i}-x_{R}\right)}^{2}\;+\;{\left(y_{i}-y_{R}\right)}^{2}\;+\;{\left(z_{i}-z_{R}\right)}^{2}}$ is the geometrical distance between the receiver and the ith satellite; $p_{R}^{⊤}=\left[x_{R},y_{R},z_{R}\right]$ and $p_{T_{i}}^{⊤}=\left[x_{i},y_{i},z_{i}\right]$ are the position coordinates of the receiver and the ith satellite, respectively; $\delta t_{r}$ and $\delta t_{i}$ are the receiver and satellite clock offsets w.r.t. the GNSS time. Since in the asymptotic region, the MLE becomes unbiased, efficient, and Gaussian distributed [36], $\epsilon_{\varrho ,i}$ and $\epsilon_{\Phi ,i}$ are zero-mean white Gaussian noise terms, and their variance is driven by the performance of $\hat{\tau }$ and $\hat{\varphi }\left(\hat{\tau }\right)$, respectively. $N_{i}$ is an ambiguous term related to the (unknown) number of phase cycles. The latter has a fractional part $B_{i}$, which depends on the initial phase of the ith satellite clock, a fractional part $B_{r}$ due to the initial phase at the receiver, and an integer part $N_{\mathrm{int},i}$, which is related to the satellite to receiver distance, then $N_{i}=B_{i}\;+\;B_{r}\;+\;N_{\mathrm{int},i}$.

### 3.3. GNSS Code-Based SPP and CRB

Standard code-based SPP exploits only the code observables in (14). If we consider *M* visible satellites being tracked, we have a complete set of code observations $y_{\varrho }^{⊤}=\left[{\hat{\varrho }}_{1},\ldots ,{\hat{\varrho }}_{M}\right]$, and the unknown parameters to be inferred are $\gamma^{⊤}=\left[p_{R}^{⊤},\;c\delta t_{r}\right]$. We can also define the noise vector as $n_{\varrho }^{⊤}=\left[\epsilon_{\varrho ,1},\ldots ,\epsilon_{\varrho ,M}\right]$, with covariance $C_{n,\varrho }$. The standard solution to this nonlinear estimation problem, $y_{\varrho }=h\left(\gamma \right)\;+\;n_{\varrho }$, is (i) to linearize the model around an initial position $p^{0}$ and (ii) use an iterative weighted least squares (WLS) estimator [38]. Considering $\delta t_{i}$ and $p_{T_{i}}$ as derived from the navigation message, the resulting linearized model can be written as:

(16) $$\begin{array}{cc} \; & \left|\right|p_{T_{i}}-p_{R}\left|\right|\approx \left|\right|p_{T_{i}}-p^{0}\left|\right|-u_{i}\left(p^{0}\right)\delta_{p},\;\delta_{p}=p_{R}-p^{0},\;u_{i}\left(p^{0}\right)=\frac{p_{T_{i}}-p^{0}}{\left|\right|p_{T_{i}}-p^{0}\left|\right|}, \end{array}$$

(17) $$\begin{array}{cc} \; & {\tilde{y}}_{\varrho }\approx \hat{H}\left(p^{0}\right)\delta \;+\;n_{\varrho },\;\delta ={\left[\delta_{p}^{⊤}\;c\delta t_{r}\right]}^{⊤},\;{\left({\tilde{y}}_{\varrho }\right)}_{i}={\hat{\varrho }}_{i}\;+\;c\delta t_{i}-\left|\right|p_{T_{i}}-p^{0}\left|\right|,\;\hat{H}\left(p^{0}\right)=\left[\begin{array}{cc} -u_{1}^{⊤}\left(p^{0}\right) & 1\\ \vdots & \vdots \\ -u_{M}^{⊤}\left(p^{0}\right) & 1 \end{array}\right], \end{array}$$

with $u_{i}\left(p^{0}\right)$ the unit steering vector to the ith satellite, evaluated at the position $p^{0}$. For the problem above, the solution can be found based on an iterative WLS adjustment (i.e., applying a Gauss–Newton method [38]), where the state δ is updated with ${\hat{d\delta }}_{WLS}$ as:

(18) $$\begin{array}{cc} {\hat{d\delta }}_{WLS} & =\arg\min_{\delta }\left\{|\right|{\tilde{y}}_{\varrho }-\hat{H}\left(p^{0}\right){\delta ||}_{W}^{2}\}={\left({\hat{H}}^{⊤}\left(p^{0}\right)W\hat{H}\left(p^{0}\right)\right)}^{-1}{\hat{H}}^{⊤}\left(p^{0}\right)W{\tilde{y}}_{\varrho }, \end{array}$$

where the optimal weighting is $W=C_{n,\varrho }^{-1}$. In addition, the CRB for the SPP problem (i.e., denoted CRB${}_{spp}$) is given by the inverse of $F_{\gamma |\gamma }\left(\gamma^{0}\right)={\hat{H}}^{⊤}\left(\gamma^{0}\right)C_{n,\varrho }^{-1}\hat{H}\left(\gamma^{0}\right)$, with $\gamma^{0}$ a selected value of γ.

### 3.4. GNSS Code/Phase-Based RTK Positioning and CRB

The previous SPP approach is limited by the time-delay MLE precision, which is directly linked to the baseband signal resolution (i.e., the shape of the ACF). Precise positioning techniques rely on the use of the signal’s phase information. Unfortunately, exploiting this information implies solving a more complicated problem, mainly because the phase measurement is ambiguous, and the estimation of the unknown number of cycles $N_{i}$ in (15) is the bottleneck [11]. As already stated, two main approaches are available in the literature: (i) differential techniques such as RTK [2] and (ii) PPP techniques [1]. It is important to notice that PPP techniques require a long convergence time and need high accuracy satellite orbits, while clock and propagation (ionospheric and tropospheric) error corrections are required, which may be obtained from a network broadcasting precise corrections. However, such corrections are not available in real time [39,40]; thus, PPP is not suitable for safety-critical applications.

In contrast, RTK exploits the connection to a reference station at a known position, which if close enough observes the same propagation errors as the receiver. Therefore, the goal is to combine such observations in order to eliminate all nuisance parameters and then estimate the base-to-receiver baseline. The combination of base and receiver observations is obtained by double-differencing, that is subtracting the measurements from the receiver w.r.t. the base station and a pivot (reference) satellite. A key point is that such a combination also eliminates the fractional phase parts $B_{i}$ and $B_{r}$; therefore, the unknown ambiguities become integer parameters. The problem of mixed real and integer parameter estimation was pioneered by Teunissen [41,42,43], and its solution typically combines a WLS with an integer least squares (ILS). It is out of the scope to give a complete discussion on RTK, and only a brief description is given in the sequel. The reader can refer to [11] and the references therein for details.

If we consider M+1 visible satellites being tracked at both the base station and the receiver, the code and phase double difference (DD) observations and the corresponding linearized model are (subscript 0 and superscript *B* are used to refer to the pivot reference satellite and the base station, respectively; superscript *R* refers to quantities related to the receiver):

(19a) $$\begin{array}{cc} \; & y_{\varrho }^{⊤}=\left[\begin{array}{c} {\hat{\varrho }}_{1,0}^{R,B},\ldots ,{\hat{\varrho }}_{M,0}^{R,B} \end{array}\right],\;{\hat{\varrho }}_{i,0}^{R,B}={\hat{\varrho }}_{i}^{R}-{\hat{\varrho }}_{i}^{B}-\left({\hat{\varrho }}_{0}^{R}-{\hat{\varrho }}_{0}^{B}\right) \end{array}$$

(19b) $$\begin{array}{cc} \; & y_{\Phi }^{⊤}=\left[\begin{array}{c} {\hat{\Phi }}_{1,0}^{R,B},\ldots ,{\hat{\Phi }}_{M,0}^{R,B} \end{array}\right],\;{\hat{\Phi }}_{i,0}^{R,B}={\hat{\Phi }}_{i}^{R}-{\hat{\Phi }}_{i}^{B}-\left({\hat{\Phi }}_{0}^{R}-{\hat{\Phi }}_{0}^{B}\right), \end{array}$$

(19c) $$\begin{array}{cc} \; & y\approx Dz\;+\;n_{\Phi ,\varrho },\;y=\left[\begin{array}{c} y_{\Phi }\\ y_{\varrho } \end{array}\right],\;z=\left[\begin{array}{c} b\\ a \end{array}\right],\;D=\left[\begin{array}{cc} B & A\\ B & 0 \end{array}\right],\;B=\left[\begin{array}{c} -{\left(u_{1}\left(p_{B}\right)-u_{0}\left(p_{B}\right)\right)}^{⊤}\\ \vdots \\ -{\left(u_{M}\left(p_{B}\right)-u_{0}\left(p_{B}\right)\right)}^{⊤} \end{array}\right], \end{array}$$

with $A=\lambda_{c}I$, B is the double-difference geometry matrix, z the set of unknown parameters, $b=p_{R}-p_{B}$ the baseline vector between the receiver and base station, and a the vector of DD integer ambiguities. The designed matrix D relates the state estimate z to the vector of observations y. Finally, the noise is:

$$\begin{array}{cc} \; & n_{\Phi ,\varrho }=\left[\begin{array}{c} n_{\Phi }\\ n_{\varrho } \end{array}\right],\;C_{n}=\left[\begin{array}{cc} C_{n_{\Phi }} & C_{n_{\Phi },n_{\varrho }}\\ C_{n_{\Phi },n_{\varrho }}^{⊤} & C_{n_{\varrho }} \end{array}\right],\;C_{n_{\left\{\Phi ,\varrho \right\}}}=\left[-1_{M,1}\;I\right]\left[\begin{array}{ccc} \sigma_{{\left\{\Phi ,\varrho \right\}}_{0}}^{2} & \; & 0\\ \; & \ddots & \;\\ 0 & \; & \sigma_{{\left\{\Phi ,\varrho \right\}}_{M}}^{2} \end{array}\right]{\left[-1_{M,1}\;I\right]}^{⊤} \end{array}$$

The solution to this problem (i.e., which has no closed-form solution because of the integer ambiguities) is typically obtained via a three-step decomposition and solved using the LAMBDA method [41,44]:

(20) $$\begin{array}{cc} \; & \underset{\hat{z}}{\underset{︸}{\left[\begin{array}{c} \hat{b}\\ \hat{a} \end{array}\right]}}=\min_{\begin{array}{c} b\in R^{3}\\ a\in Z^{M} \end{array}}{∥y-D\left[\begin{array}{c} b\\ a \end{array}\right]∥}_{C_{n}}^{2}=\min_{\begin{array}{c} b\in R^{3}\\ a\in R^{M} \end{array}}{∥y-D\left[\begin{array}{c} \bar{b}\\ \bar{a} \end{array}\right]∥}_{C_{n}}^{2}\;+\;\min_{a\in Z^{M}}{∥\bar{a}-a∥}_{C_{\bar{a}}}^{2}\;+\;\min_{b\in R^{3}}{∥\bar{b}|a-b∥}_{C_{\bar{b}|a}}^{2}. \end{array}$$

We can identify: (i) a first WLS problem where the integer nature of the ambiguities is disregarded, the so-called float solution, (ii) a second ILS [11], for which an integer ambiguity solution is obtained, and (iii) a third WLS refinement or correction step, the so-called fixed solution. The CRB associated with the float solution, the so-called CRB${}_{real}$, is given by the inverse of $F_{z|z}\left(z^{0}\right)=D^{⊤}C_{n}^{-1}D$. The mixed real/integer CRB associated with the fixed solution, the so-called CRB${}_{real/integer}$, was discussed in [45].

## 4. GNSS Meta-Signal and HO-BOC Signals Delay/Phase Estimation Results

In the problem of interest, the first step of the receiver (see Section 3.1) is the time-delay and phase ML estimation. Therefore, in order to assess the performance for the different GNSS meta-signals and HO-BOC signals in Section 2, we compared the delay and phase MLEs (9) and (10) with the corresponding CRBs in (11) and (12). In the rest of the article, we consider the following sampling frequencies:

- Galileo E5 AltBOC(15,10)—$F_{s}=60$ MHz.
- GPS L2C - L5Q AltLOC(25,10,1,75)—$F_{s}=75$ MHz.
- Galileo E5BQ + E6B AltLOC(35,10,5,112)—$F_{s}=112$ MHz.
- BeiDou B2AQ + B3Q AltLOC(45,10,10,120)—$F_{s}=120$ MHz.
- Galileo E5AQ + E6B AltLOC(50,10,5,132)—$F_{s}=133$ MHz.
- BeiDou B2 + B3Q AltLOC(37.5,C${}_{B2}\left(t\right)$,10,125)—$F_{s}=125$ MHz.
- Galileo E5 + E6B AltLOC(42.5,C${}_{E5}\left(t\right)$,5,132)—$F_{s}=135$ MHz.
- GPS L1 - M BOCsin(10,5)—$F_{s}=30$ MHz.
- Galileo E1 PRS BOCcos(15,2.5)—$F_{s}=40$ MHz.
- Galileo E6 PRS BOCcos(10,5)—$F_{s}=30$ MHz.
- BeiDou B1A BOCcos(14,2)—$F_{s}=40$ MHz.

**Figure 5:**
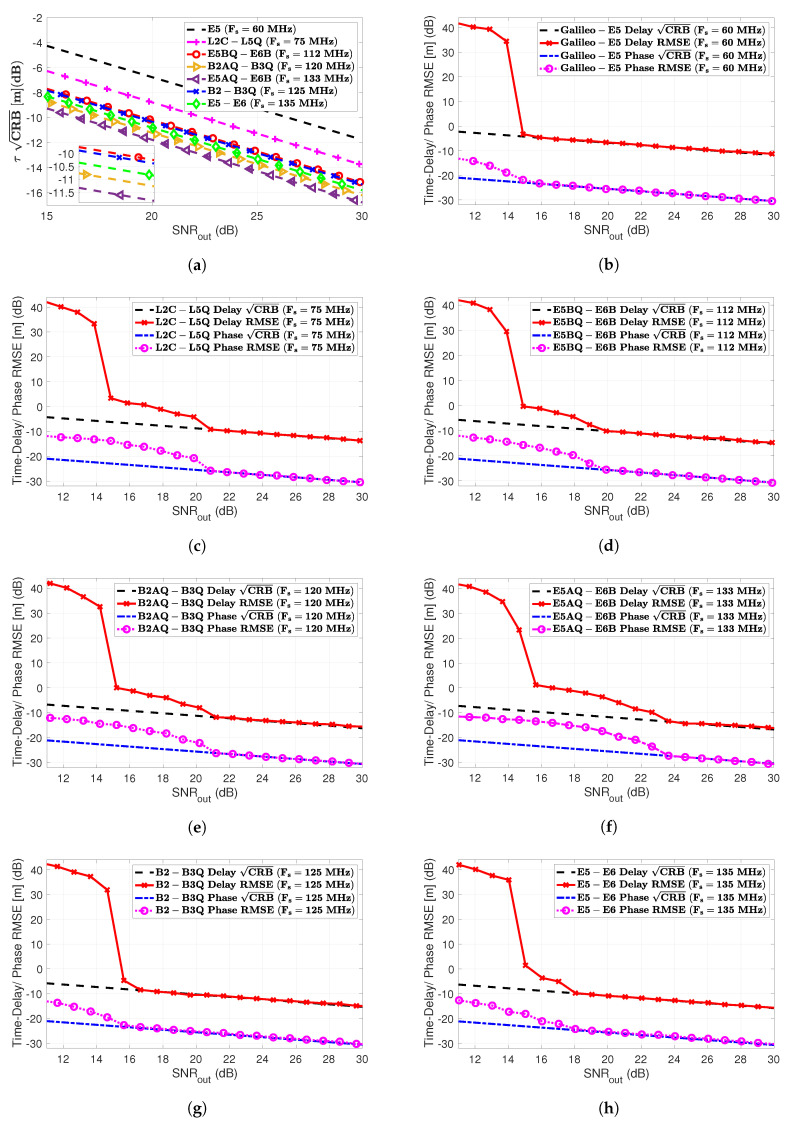
The comparison of the different time-delay CRBs (m) is shown in Subplot (**a**). Time-delay and phase RMSE (m) and the corresponding CRBs (m) for the different GNSS meta-signals are shown in Subplots (**b**) Galileo E5, (**c**) GPS L2C + L5Q, (**d**) Galileo E5B + E6B, (**e**) BeiDou B2AQ + B3Q, (**f**) Galileo E5A + E6B, (**g**) BeiDou B2 + B3Q and (**h**) Galileo E5 + E6B.

The MLEs and CRBs were computed considering $\alpha =\left(1\;+\;j\right)\cdot \sqrt{SNR_{in}/2}$, where *j* represents the imaginary number. The root mean squared error (RMSE) for the MLE was obtained from 1000 Monte Carlo runs. Notice that the SNR${}_{\mathrm{out}}$ in the following results refers to the SNR at the output of the MLE, and the maximum is given by:

(21) $$\begin{array}{cc} \; & {\mathrm{SNR}}_{\mathrm{out}}=\frac{F_{s}{\left|\alpha \right|}^{2}c^{H}c}{\sigma_{n}^{2}}=\frac{C}{N_{0}}T_{\mathrm{PRN}}L_{c}, \end{array}$$

where $C/N_{0}$ (dB-Hz) is the carrier-to-noise density ratio, $T_{\mathrm{PRN}}$ is the single code duration, $L_{c}$ is the number of codes, and therefore, $T_{I}=T_{\mathrm{PRN}}\times L_{c}$ is the coherent integration time. Then, we could verify that SNR${}_{\mathrm{out}}=25$ dB and $T_{I}=10$ ms implied a $C/N_{0}=45$ dB-Hz, which was a nominal GNSS value. The results for the different meta-signals considered in this article are summarized in Figure 5.

- First, notice from Subplot (a) that there was a difference of: (i) 2 dB between E5 and L2 + L5 and (ii) 1.44 dB between L2 + L5 and E5B + E6. Among the rest of the meta-signals, the maximum difference of 1.6 dB was exhibited between E5B + E6 and E5A + E6. Therefore, overall and after the convergence of the delay MLE, we had a maximum factor equal to three among meta-signals.
- Considering SNR${}_{\mathrm{out}}$ = 25 dB as a reference value, the time-delay standard deviation was (i.e., following the order in Figure 5a) roughly (results in cm): 12, 7.5, 5.4, 4.2, 3.7, 5.3, and 4.6.
- What was more interesting was the impact of the subcarrier, i.e., the ACF, into the time-delay CRB values and the MLE convergence. Comparing the results in Figure 5 with the corresponding ACFs in Figure 2, it was clear that:
  (1) a narrower ACF main peak implied better time-delay estimation capabilities (see Subplots (a) and (b) in Figure 2), i.e., a lower CRB,
  (2) larger secondary peaks and closer to the main one directly impacted the convergence to the CRB and the ML threshold. This was directly related to possible false locks, which were more probable at lower SNR when the ACF secondary peaks were large and close to the main one of interest. For instance, for Galileo E5, E5 + E6 and B2 + B3, these secondary peaks (see Subplots (g) and (h) in Figure 2) only slightly affected the convergence to the CRB, which was reached for SNR${}_{\mathrm{out}}$ = [16–18] dB. In contrast, larger secondary peaks in L2 + L5, B2A + B3, E5B + E6, and E5A + E6 led to a convergence in SNR${}_{\mathrm{out}}$ = [20–24] dB.
- Regarding the phase CRB and MLE, the latter converged only if the corresponding time-delay MLE did. This was because the phase MLE (10) was given by the argument evaluated at the time-delay MLE. Therefore, as discussed above for the time-delay, large secondary peaks directly degraded the convergence to the CRB, which would have an impact on carrier phase-based positioning techniques (see Section 6). It is important to notice also the performance gain around 15 dB (a factor of 30) between delay and phase estimates.

We could conclude that in terms of robustness (i.e., being able to operate at lower SNRs), the best choices were Galileo E5 and the combinations with the full-bandwidth E5 and B2, i.e., E5 + E6 and B2 + B3.

**Figure 6:**
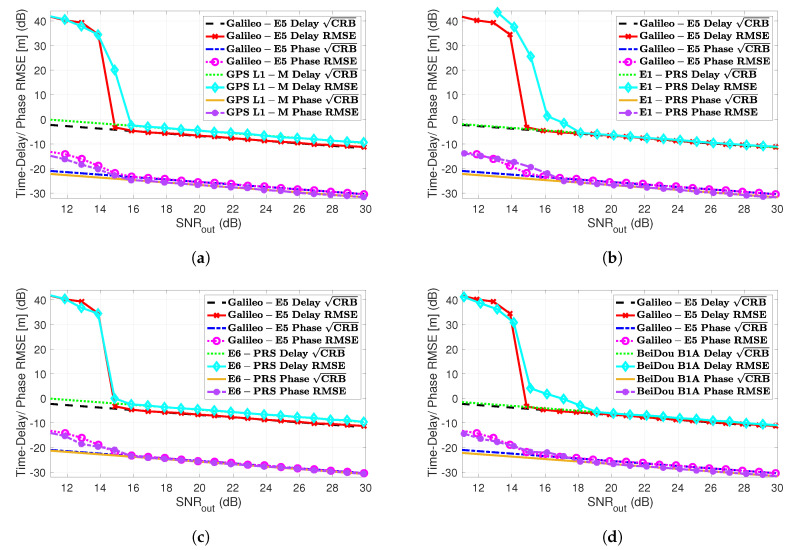
Time-delay/phase RMSE and CRBs (m) for different GNSS HO-BOC signals: (**a**) GPS L1-M BOCsin(10,5), (**b**) Galileo E1-PRS BOCcos(15,2.5), (**c**) Galileo E6-PRS BOCcos(10,5) and (**d**) BeiDou B1A BOCcos(14,2)

The time-delay/phase MLEs and the corresponding CRBs results for the different HO-BOC signals are summarized in Figure 6. The results were also compared to the MLEs and CRB obtained with Galileo E5. Taking into account the ACF shape in Figure 4, again, the impact of the secondary peaks was clear, which induced false locks and therefore a convergence degradation, which translated to a MLE threshold behavior that was shifted to the right. This effect was similar to the one discussed for the GNSS meta-signals, shown in Figure 5. For the HO-BOC signals considered, we can point out the following:

- As already anticipated, the achievable performance for the time-delay estimation, with the E1-PRS and the B1A signals, was almost the same compared to Galileo E5. This was determined by the shape of the ACF main peak and the signal bandwidth considered.
- Because of the much larger secondary peaks of the E1-PRS and the B1A signals, which were roughly located at the same position, the convergence region to the CRB was degraded w.r.t. the Galileo E5 signal (i.e., [2–3] dB).
- Regarding the L1-M and E6-PRS signals, because the ACF main peaks were wider, then the time-delay CRB was slightly degraded compared to E5 (i.e., roughly 2 dB).
- Because the secondary peaks of the L1-M and E6-PRS signals were further apart from the main one and their value was not extremely large, w.r.t. Galileo E5, the convergence region was not affected.
- Again, as for the different GNSS meta-signals, the convergence of the phase MLE was driven by the convergence of the time-delay MLE; therefore, only the L1-M and E6-PRS signals provided a performance similar to the Galileo E5 signal.

We could conclude that in terms of robustness and with only a slight performance degradation w.r.t. the Galileo E5 signal, the best choices were the L1-M and E6-PRS HO-BOC signals (i.e., BOCsin(10,5) and BOCcos(10,5)). In the results provided, notice that these two signals used a sampling frequency $F_{s}=30$ MHz, which was half of the bandwidth exploited for Galileo E5, i.e., using $F_{s}=60$ MHz.

## 5. GNSS Meta-Signal SPP Performance Results

**Figure 7:**
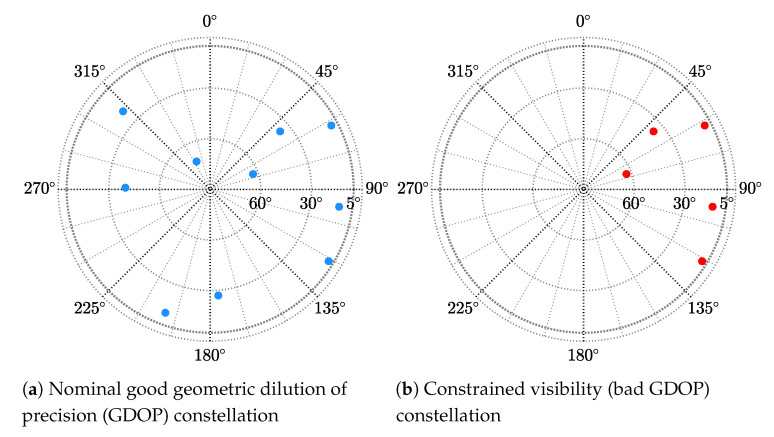
Satellite constellation skyplots for both nominal (**a**) and non-nominal conditions (**b**).

In the previous Section 4, we assessed the achievable performance limits for time-delay and phase estimation, considering a set of representative meta-signals. In the sequel, we assess how the previous time-delay estimates translate into the position domain considering the standard WLS SPP solution in Section 3.3. Notice that the covariance of the code observables noise $C_{n,\varrho }$ was set according to the corresponding MLE precision. We considered two satellite constellation scenarios, with a good and bad geometric dilution of precision (GDOP), respectively, which are shown in Figure 7.

(1) Nominal conditions with a good GDOP (Subplot (a) in Figure 7).
(2) Constrained satellite visibility, or bad GDOP (Subplot (b) in Figure 7). Even if it is out of the scope to consider realistic multipath channels, this scenario was representative of an urban canyon situation, where part of the sky may be blocked by buildings.

Without loss of generality and with the aim to determine the ultimate performance w.r.t. the SNR at the output of the estimator, we considered that all satellites were received with the same power. For instance, if the high-elevation satellites were received with a $C/N_{0}$ around 45 dB-Hz, the low elevation satellites with a $C/N_{0}$ around 40 dB-Hz, and the receiver used a coherent integration time $T_{I}=10$ ms, this would translate into an ultimate SPP performance in the range SNR${}_{\mathrm{out}}$ = [20–25] dB.

**Figure 8:**
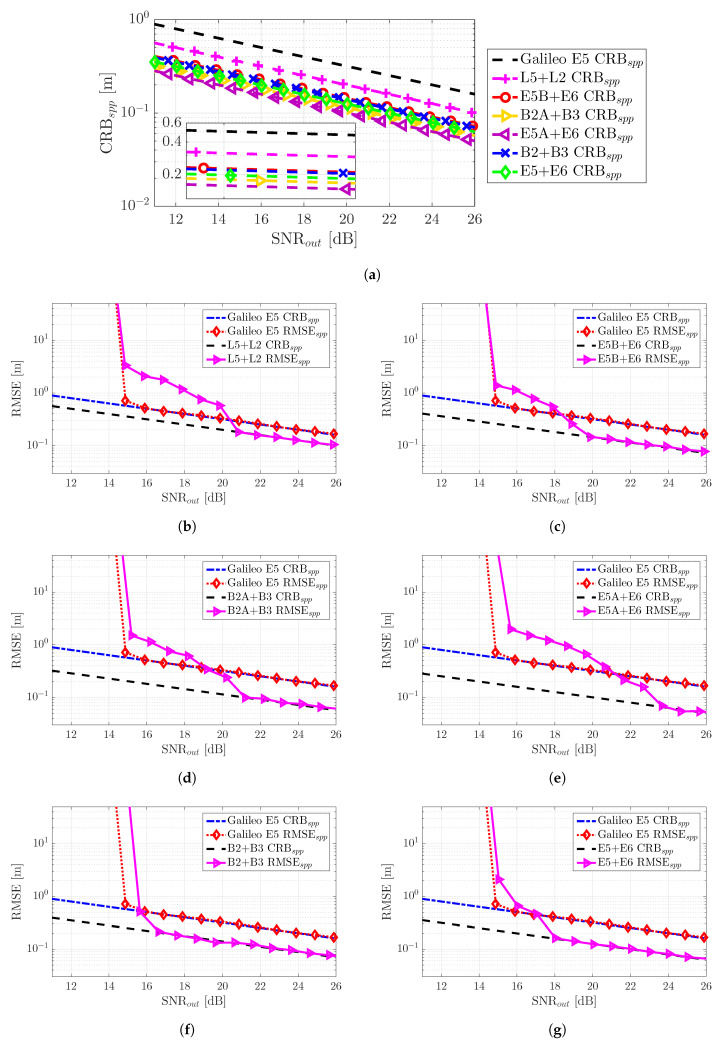
The comparison of the different SPP CRBs (m) is shown in Subplot (**a**). Nominal good GDOP scenario SPP RMSE (m) and the corresponding CRBs (m) for the different GNSS meta-signals are shown in Subplots: (**b**) GPS L2C + L5Q, (**c**) Galileo E5B + E6B, (**d**) BeiDou B2AQ + B3Q, (**e**) Galileo E5A + E6B, (**f**) BeiDou B2 + B3Q and (**g**) Galileo E5 + E6B.

The SPP performance results (i.e., position RMSE) for the good GDOP scenario are summarized in Figure 8. First, notice that the CRBs are shown together for the different meta-signals in Subplot (a). We can easily identify the same tendency as in Figure 5a, and as expected, better time-delay estimation performance implied a better position estimate.

- Again, if we considered SNR${}_{\mathrm{out}}=25$ dB as a reference value, the position standard deviation was (i.e., following the order in Figure 8a) roughly: 18 cm, 11 cm, 8 cm, 6.5 cm, 5.5 cm, 8 cm, and 7 cm. Therefore, w.r.t. the time-delay results at SNR${}_{\mathrm{out}}=25$ dB previously discussed, the geometry matrix induced a slight performance degradation.
- Any of the meta-signals could be considered as a precise code-based positioning alternative.
- Obviously, the convergence time due to false locks into secondary peaks of the ACF directly translated to the corresponding convergence into the position domain CRB. This further supported the fact that in terms of SNR robustness, the best choices were Galileo E5 and the combinations with the full-bandwidth E5 and B2, i.e., E5 + E6 and B2 + B3. The slight performance improvement obtained with the other signal combinations was not worth the threshold degradation.

**Figure 9:**
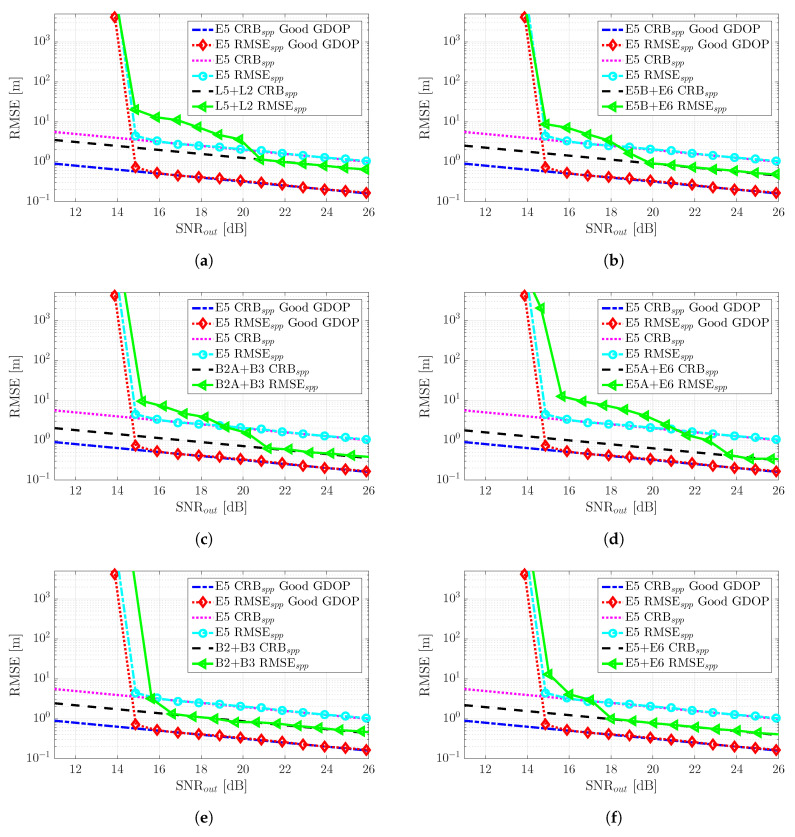
Non-nominal bad GDOP scenario SPP RMSE (m) and the corresponding CRBs (m) for the different GNSS meta-signals: (**a**) GPS L2C + L5Q, (**b**) Galileo E5B + E6B, (**c**) BeiDou B2AQ + B3Q, (**d**) Galileo E5A + E6B, (**e**) BeiDou B2 + B3Q and (**f**) Galileo E5 + E6B. The RMSE/CRB SPP performance for Galileo E5 under nominal good GDOP conditions is shown for comparison.

The SPP performance results for the bad GDOP constrained satellite visibility scenario are summarized in Figure 9. The previous results with a good GDOP for Galileo E5 are shown as a reference. Overall, we can see that considering a bad GDOP only degraded the performance, but did not change the behavior of the CRBs and MLEs. The positioning standard deviation performance results for the reference value SNR${}_{\mathrm{out}}=25$ dB were as follows (i.e., recall that the E5 nominal performance in the previous scenario was 18 cm): (i) E5, 1.15 m; (ii) L2 + L5, 69 cm; (iii) E5B + E6, 52 cm; (iv) B2A + B3, 40 cm; (v) E5A + E6, 32 cm; (vi) B2 + B3, 51 cm; and (vii) E5 + E6, 44 cm. Notice that the performance degradation was remarkable; therefore, in some safety-critical applications operating in harsh propagation conditions, meta-signals may not provide the precision needed.

In conclusion, as for the time-delay estimation and regardless of the satellite geometry, the best compromise in terms of robustness, performance, and estimator behavior was provided by Galileo E5 and the full-bandwidth E5 + E6 and B2 + B3 combinations, meta-signals providing better performance, but requiring a double bandwidth.

## 6. GNSS Meta-Signal RTK Performance Results

So far, we have discussed the ultimate time-delay and phase MLE performance obtained with both meta-signals and HO-BOC signals and the meta-signal-based SPP performance considering both good and bad satellite geometries. The remaining open point is the impact of the phase MLE behavior on positioning techniques that exploit such phase information. To address this point, we considered the RTK positioning problem in Section 3.4. Again, we considered the two scenarios depicted in Figure 7. In this case and taking into account the three-step RTK solution in (20), we wanted to assess the performance of the float solution (denoted as RMSE${}_{real}$), the fixed solution considering all the estimates (denoted as RMSE${}_{mixed}$), and the fixed solution considering only the estimates that were declared as a correct ambiguity fix (denoted as RMSE${}_{correct\;amb}$), the latter only shown for the bad GDOP scenario. The position RMSEs for the RTK performance were obtained from $10^{4}$ Monte Carlo realizations.

### 6.1. Nominal Conditions: Good Satellite Geometry Scenario

**Figure 10:**
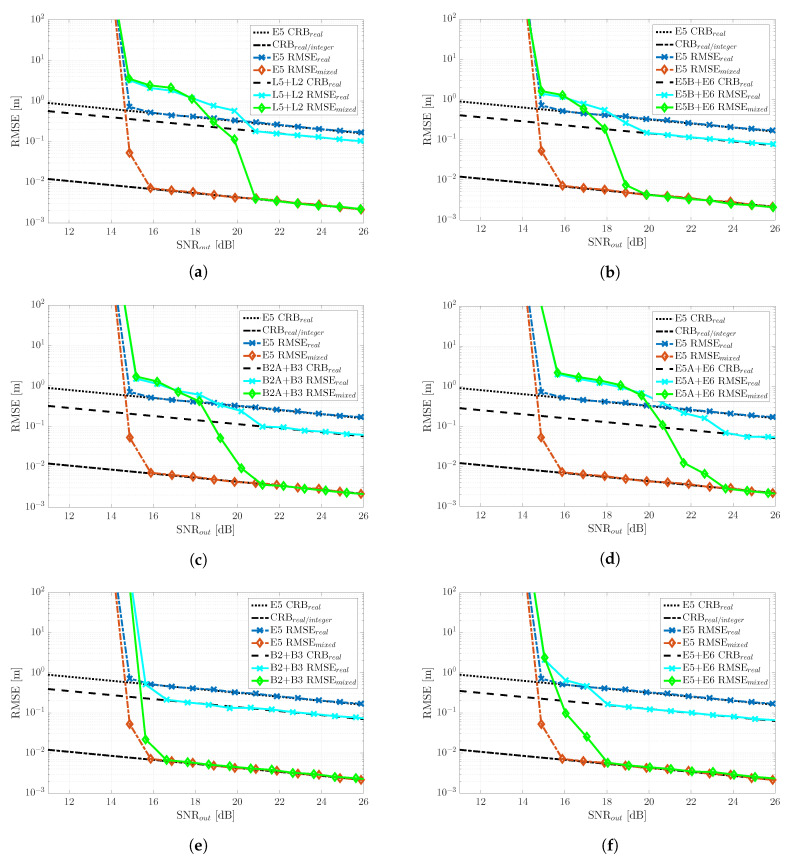
Nominal good GDOP scenario RTK RMSE (m) and the corresponding CRBs (m) for the different GNSS meta-signals: (**a**) GPS L2C + L5Q, (**b**) Galileo E5B + E6B, (**c**) BeiDou B2AQ + B3Q, (**d**) Galileo E5A + E6B, (**e**) BeiDou B2 + B3Q and (**f**) Galileo E5 + E6B.

**Figure 11:**
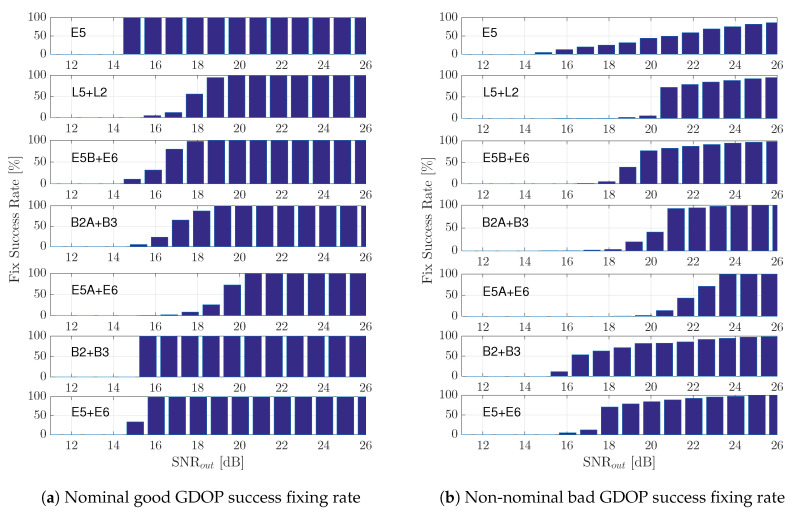
Nominal (**a**) and non-nominal (**b**)—good and bad GDOP, respectively—scenario RTK fixing success rate.

The RTK performance results for the good GDOP scenario are summarized in Figure 10. The first thing to point out is the huge performance gain provided by a correct exploitation of the phase information, which was clear from the gap between CRB${}_{real}$ and CRB${}_{real/integer}$. For instance, considering the reference Galileo E5 signal and a SNR${}_{\mathrm{out}}=25$ dB, CRB${}_{real}=18$ cm, and CRB${}_{real/integer}=2$ mm. Several interesting conclusions can be extracted from these results:

- The convergence to the RTK CRB${}_{real/integer}$ was driven by the phase MLE threshold region, that is for every meta-signal, the RMSE${}_{mixed}$ (i.e., fixed solution considering all the estimates and not only the correct fix ones) started to deviate from the corresponding CRB at the same point as the phase estimate deviated from the phase CRB, which in turn was driven by the time-delay MLE behavior. Recall that such slow convergence (or intermediate threshold behavior) was directly related to the false locks due to large secondary peaks. Therefore, a first conclusion was that subcarriers that induced large secondary ACF peaks strongly impacted the achievable RTK performance.
- The second interesting point was that when the RMSE${}_{mixed}$ started to deviate from the CRB, it rapidly joined thefloat solution behavior (i.e., RMSE${}_{real}$). Therefore, it was fundamental to characterize the threshold region correctly. Below such SNR${}_{\mathrm{out}}$, there was no reason to try to fix the phase ambiguities, and therefore, a straight WLS could be used instead. This supported the statement that RTK typically does not work in harsh propagation conditions, at least for some of the signals.
- The impact of the two previous points can also be seen on the fixing success rate, which is shown in Figure 11. In the results presented in Figure 10, only when the success rate was equal to 100%, the RMSE${}_{mixed}$ was on CRB${}_{real/integer}$. When the noise increased and the fixing probability decreased, the RMSE${}_{mixed}$ rapidly deviated from the optimal.

Overall, because the goal was to maximize the operation region correctly exploiting the phase information, the best performance was provided by the E5 signal, for which the threshold was SNR${}_{\mathrm{out}}=16$ dB. Therefore, we could conclude that in the nominal RTK case, there was no sense in exploiting GNSS meta-signals (i.e., except E5). In addition, among the meta-signals, E5 + E6 and B2 + B 3 provided a clear advantage w.r.t. the other signal combinations.

### 6.2. Non-Nominal Conditions: Constrained Satellite Visibility (Bad GDOP) Scenario

**Figure 12:**
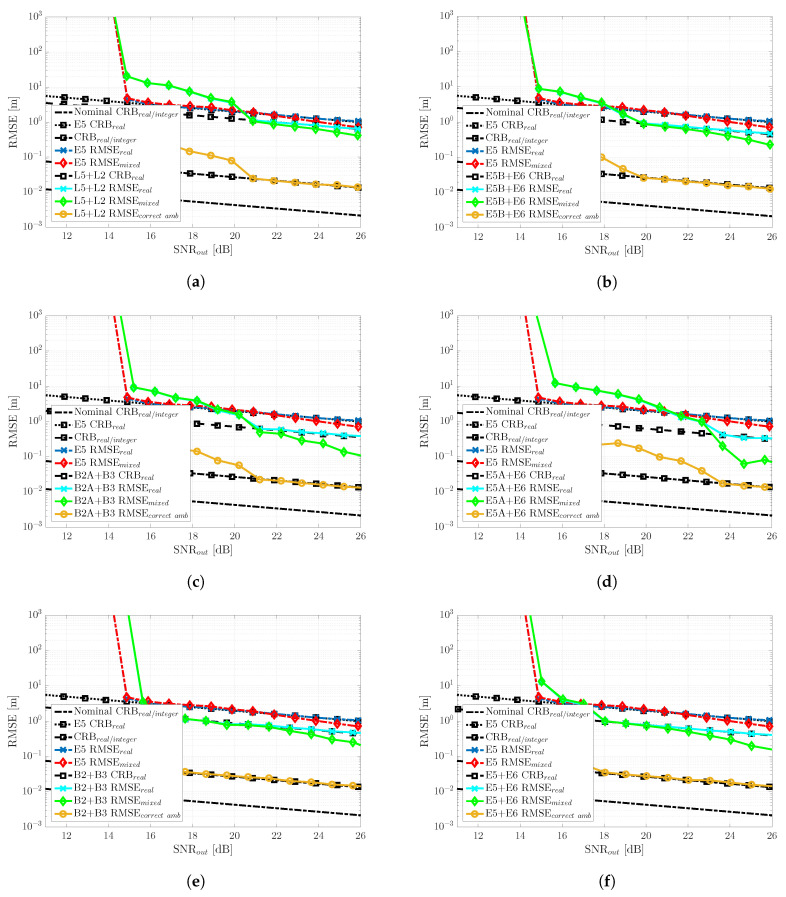
Non-nominal bad GDOP scenario RTK RMSE (m) and the corresponding CRBs (m) for the different GNSS meta-signals: (**a**) GPS L2C + L5Q, (**b**) Galileo E5B + E6B, (**c**) BeiDou B2AQ + B3Q, (**d**) Galileo E5A + E6B, (**e**) BeiDou B2 + B3Q and (**f**) Galileo E5 + E6B.

The RTK performance results for the constrained satellite visibility scenario are summarized in Figure 12. In this case, we can point out the following:

- First, notice the performance degradation on the achievable RTK performance, that is between the nominal CRB${}_{real/integer}$ (good GDOP scenario) and the corresponding one for the current bad GDOP case. At SNR${}_{\mathrm{out}}=25$ dB. the former was CRB${}_{real/integer}=2$ mm (nominal) and the latter CRB${}_{real/integer}=1.5$ cm (non-nominal).
- With respect to the nominal case where the best performance was obtained with Galileo E5, in the bad GDOP scenario and for the range of SNR considered, using this signal barely improved the float solution for SNR${}_{\mathrm{out}}>24$ dB. Therefore, in this case, the geometry matrix had a strong impact on the ILS compared to the WLS results in Figure 9. This result came from a drastic drop of the fix success rate, as shown in Figure 11. A wrong fix could completely spoil the solution, and a 100% fix was needed for RMSE${}_{mixed}$ to be on CRB${}_{real/integer}$. However, notice that compared to L2 + L5, E5B + E6, E5A + E6, or B2A + B3, where the threshold effect was more severe, considering E5 was still useful in the range SNR${}_{\mathrm{out}}$ =[16–20] dB. However, there was no need to try to exploit the phase and fix the ambiguities; therefore, the user could directly keep a code-based RTK solution.
- For high SNR regimes (i.e., SNR${}_{\mathrm{out}}>22$ dB), we could see the impact of the time-delay precision, which allowed RMSE${}_{mixed}$ < RMSE${}_{real}$. This was clear looking at the E5A + E6 and B2A + B3 signals, which provided the lowest CRBs (see Figure 8a). However, in any case, the performance improvement was not worth the estimator behavior at lower SNRs.
- Again, only the full-bandwidth combinations E5 + E6 and B2 + B3 provided a consistent performance, improving the Galileo E5, together with the highest success ratio, as shown in Figure 11. Considering that we had an ambiguity fixing measure, these signals provided the best trade-off, when the performance was fixed, on CRB${}_{real/integer}$ as given by the RMSE${}_{correct\;amb}$, and when not fixed, the performance was equal to or better than the float solution (i.e., RMSE${}_{mixed}\leq$ RMSE${}_{real}$).
- It is also worth pointing out that the behavior of RMSE${}_{correct\;amb}$ for all the meta-signals except E5 + E6 and B2 + B3 was due to the very low fix success ratio (i.e., the worst one being E5A + E6), which implied that very few realizations from the $10^{4}$ Monte Carlo runs were averaged. That was the reason why the RMSE with a correct ambiguity fix was not always on CRB${}_{real/integer}$.

As a conclusion, even if in the previous good GDOP scenario, the best choice was Galileo E5, it was clear that regardless of the satellite geometry, the best performance and robustness tradeoff was provided by the full-bandwidth Galileo E5 + E6 and BeiDou B2 + B3 combinations, the latter being slightly better. In addition, for these two signals, the fixing success rate was maximized.

**Figure 13:**
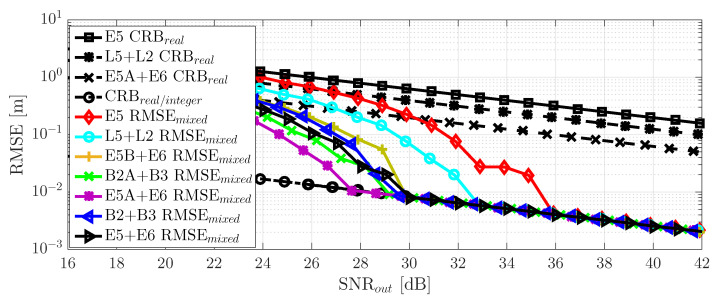
Non-nominal bad GDOP scenario RTK RMSE/CRB (m) convergence at high SNR.

To conclude the meta-signal-based RTK positioning performance analysis, it was interesting to assess which was the convergence point of the different meta-signals in a bad GDOP scenario; in other words, when RMSE${}_{mixed}$ = CRB${}_{real/integer}$ and, thus, the method correctly exploited the phase information. This result is shown in Figure 13, where the most remarkable point was that the convergence of the MLE to CRB${}_{real/integer}$ was driven by the time-delay estimation precision. A lower CRB${}_{real}$ (i.e., better time-delay estimation precision) implied a faster convergence to CRB${}_{real/integer}$ (i.e., the threshold region at a lower SNR). In this case, the threshold was in the range SNR${}_{\mathrm{out}}=\left[28-36\right]$ dB, the best case obtained with E5A + E6 and the worst one by Galileo E5, which provided the best performance under nominal conditions.

Taking into account these convergence results, the performance ones in Figure 12 and the success rate in Figure 11, further confirmed that the best choices were the Galileo E5 + E6 and BeiDou B2 + B3 combinations.

## 7. GNSS HO-BOC Signals vs. Galileo E5 Positioning Performance Results

**Figure 14:**
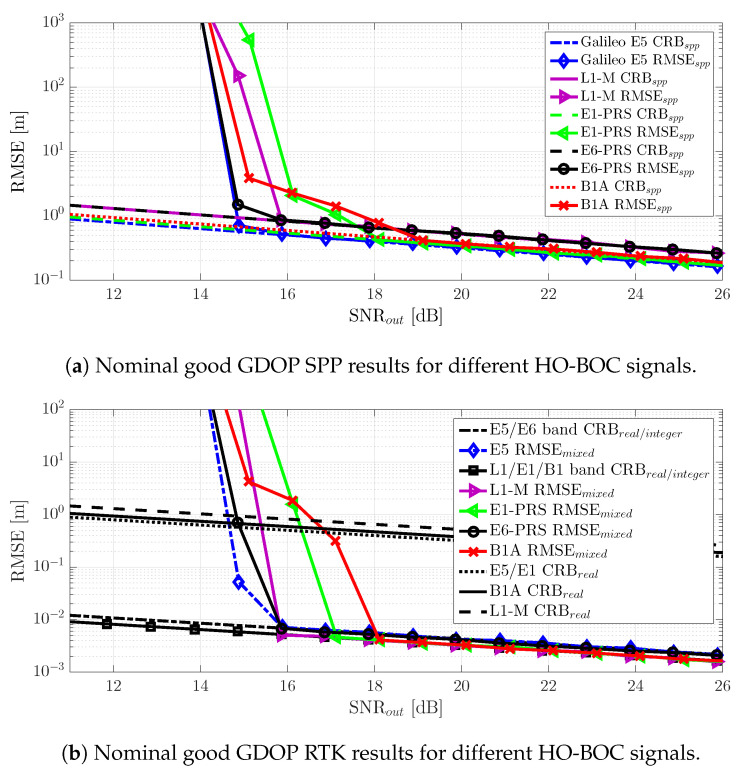
CRB and RMSE for the SPP (**a**), CRB and RMSE for RTK (**b**) under nominal conditions and for different HO-BOC signals. The RMSE/CRB SPP and RTK performance for Galileo E5 are shown for comparison.

Finally, to complete the discussion, we summarize in Figure 14 the SPP and RTK positioning results using the four HO-BOC signals (see Section 2.5), which were compared against the Galileo E5 signal performance. We only show the results for a good satellite geometry because in the bad GDOP case, the conclusions were the same as for the Galileo E5 signal in Section 6.2.

- First, notice that for the SPP, the same time-delay threshold behavior was obtained, the L1-M and E6-PRS HO-BOC signals being the ones that provided a the best robustness with a slight performance degradation w.r.t. E5. The E1-PRS and B1A signals achieved a similar E5 asymptotic performance, but needed the better [3–4] dB of SNR at the output of the MLE.
- In the RTK positioning case, CRB${}_{real/integer}$, which mainly depended on the carrier signal wavelength $\lambda_{c}$, was slightly better for the HO-BOC signals, which operated at a higher frequency in the L1/E1/B1 band. Under nominal conditions, this implied that the carrier phase-based solution was improved by the L1-M and E6-PRS signals, because they preserved the same threshold behavior. In contrast, as for the SPP case, the threshold for the E6-PRS and B1A signals was degraded [1–2] dB.
- Notice that for a bad GDOP and because the RTK convergence was driven by the time-delay estimation performance (refer to Figure 13), the four HO-BOC signals behaved similar to or worse than the Galileo E5.

We could conclude that the L1-M and E6-PRS signals are recommended, in scenarios with bad satellite geometries for SPP and for nominal conditions in the RTK case. In the latter case and considering an SPP solution, the E1-PRS and B1A signals provided a performance similar to Galileo E5.

## 8. Conclusions and Outlook

In this contribution, we provided a comprehensive analysis of the achievable positioning performance limits, for both code-based SPP and carrier phase-based RTK approaches, using the complete set of GNSS meta-signals and some representative HO-BOC signals. The analysis was conducted by resorting to previously derived CRB expressions, which depended only on the signal samples and the corresponding MLE. First, the time-delay and phase MLE performance was assessed, which in turn drove the position RMSE behavior. Several remarkable conclusions were drawn throughout the article, summarized in the sequel:

- Time-delay/phase estimation: Regarding the time-delay estimation, while the achievable precision was linked to the ACF main peak width, the MLE threshold behavior was driven by the secondary peaks of the ACF. This implied that signals with large secondary peaks close to the main one did not converge directly to the CRB, but had a transitory region, therefore being less robust to SNR variations. From the set of meta-signals, only the combinations with the full-bandwidth B2 and E5 signals, that is the combination of AltBOC(15,10) with the signals in the B3 and E6 bands, respectively, exhibited low secondary peaks and minimized the impact on the MLE threshold. It was also shown that using much larger bandwidth combinations with signals in the L1/E1/B1 band was not worthwhile because this induced larger secondary peaks. In terms of phase estimates, the performance was directly linked to the corresponding time-delay MLE, therefore following the same threshold behavior. In terms of robustness and performance, the best choices were E5 + E6 and B2 + B3. For the set of HO-BOC signals considered: (i) the achievable time-delay performance with E1-PRS and B1A was similar to E5, but with larger secondary peaks, which worsened the threshold behavior; and (ii) the time-delay achievable performance with the L1-M and E6-PRS signals was slightly worse compared to E5, but without threshold degradation.
- SPP: As for the time-delay estimation, it was shown that regardless of the satellite geometry, the best compromise in terms of robustness, performance, and estimator behavior was given by Galileo E5 and the full-bandwidth E5 + E6 and B2 + B3 combinations, the meta-signals providing greater precision, but requiring double the bandwidth. If HO-BOC signals were considered: (i) the E1-PRS and B1A signals are recommended for nominal conditions, achieving a performance similar to Galileo E5 with half the bandwidth; and (ii) the L1-M and E6-PRS signals are recommended for bad satellite geometries.
- RTK: Under nominal conditions, it was shown that it made no sense to exploit GNSS meta-signals as the Galileo E5 signal already provided the best achievable performance. These results were slightly improved if using L1-M and E6-PRS HO-BOC signals. For RTK positioning under bad satellite visibility conditions it was shown that the convergence of the Galileo E5 signal was significantly worsen; therefore, regardless of the satellite geometry, the best performance and robustness trade-off was provided by the full-bandwidth Galileo E5 + E6 and BeiDou B2 + B3 combinations, the latter being slightly better. In addition, for these two signals, the fixing success rate was maximized. In the HO-BOC-based RTK under non-nominal conditions, as for E5, both signals provided poor performances.

As a final remark, the following recommendations could be extracted from this article: (i) if the receiver is constrained by the signal bandwidth and the user has access to restricted codes, it is recommended to use the E1-PRS or B1A signal under nominal conditions, and L1-M or E6-PRS under non-nominal conditions; (ii) if the receiver is able to operate at 60 MHz, it is recommended to exploit the full-bandwidth Galileo E5 signal; and (iii) in terms of robustness and performance, if the receiver can operate at 135 MHz, the best choice is to use the GNSS meta-signals E5 + E6 or B2 + B3. These meta-signals provided the best overall performances regardless of the positioning method used, the satellite constellation geometry, or the propagation conditions.

It is worth noting that the present article provided the ultimate achievable performance using HO-BOC and GNSS meta-signals, but further analysis could be conducted: (i) performance loss w.r.t. the optimal under multipath conditions; (ii) the impact of high-dynamic conditions; (iii) coherent vs. non-coherent architectures in very weak signal conditions; (iv) analysis w.r.t. external errors such as ionospheric/tropospheric delays, orbital or satellite clock errors; or (v) a specific receiver design to avoid false locks to secondary ACF peaks or meta-signal tracking strategies.
